# Nutrient utilization and rumen metabolism in sheep fed *Prosopis juliflora* pods and Cenchrus grass

**DOI:** 10.1186/2193-1801-2-598

**Published:** 2013-11-09

**Authors:** Om Hari Chaturvedi, Artabandhu Sahoo

**Affiliations:** Division of Animal Nutrition, Central Sheep and Wool Research Institute, Avikanagar, Rajasthan 304 501 India

**Keywords:** *Prosopis juliflora* pods, Cenchrus grass, Nutrient utilization, Rumen metabolism, Sheep

## Abstract

The present study was conducted to evaluate the effect of replacement of *Prosopis juliflora* pods (PJP) in the concentrate mixture fed to sheep along with Cenchrus grass (CG). Twenty four adult Malpura rams (3–5 years, 45.4 ± 4.26 kg) were randomly divided into three equal groups on the basis of age and live weight (LW). Mature green *Prosopis juliflora* pods were collected, dried and ground to replace concentrate mixture at 0 (G1), 30% (G2) or 40% (G3). Cenchrus grass was offered *ad libitum* whereas the concentrate mixture was fed at the rate of 1% LW. Nutrient digestibility did not differ among the groups while N balance data showed reduced utilization in G3. However, the animals in G1, G2 and G3 maintained LW and their nutritional profile indicated surplus of energy and protein with comparable feed value of all the three diets. Rumen pH decreased and volatile fatty acids increased (P < 0.05) 4 h after feeding in G2 and G3 compared to G1. In summary, *Prosopis juliflora* pods can replace concentrate mixture up to 40% in sheep feeding without any adverse effect on nutrient intake and utilization as well as rumen fermentation characteristics.

## Introduction

In many countries including India, sheep are raised on rangeland and stubble grazing. Fodder trees and bushes known as top feeds often become part of the diet during scarcity (lean) season to make up the scarce vegetation to meet with nutritional requirements (Rangnekar 2002). The rangelands of the semi-arid regions of India are covered with a wide variety of vegetation mainly grasses, bushes, shrubs and trees and the biomass yield of the community rangelands is generally low due to high stocking density and overgrazing (Sankhyan et al. 1999). Therefore, sheep grazing on such land are underfed for most part of the year. Further, a majority of the sheep farmers in this region do not supplement concentrate even in critical physiological stages thereby undermining average production from sheep (Chaturvedi et al. 2008). In most of the tropical countries, the rangeland area is gradually shrinking that adds to stocking pressure and low productivity. Consequently, in this scenario of feed resource crunch, expanding use of alternate feeds and fodder seems to be one of the viable approaches.

*Prosopis juliflora* Swartz (family Leguminosae, sub-family Mimosoideae) is a perennial, fast-growing, often ever-green and drought resistant shrub or tree that grows in semi-arid areas all over the world and is commonly known as Vilayati babool in India. It has also been planted successfully under desert like conditions where it is often used to halt shifting sand dune encroachment. A high yield (169 kg/tree/year) with production output estimated over two million tonnes in India (Sawal et al. 2004), *P. juliflora* pods (PJP) promises to be an alternate feed resource that can be used by feed processing industries for livestock. Usefulness of PJP in livestock feeding has been reported earlier (Sawal et al. 2004; Singh et al. 2004; Pandya et al. 2005; Sharma et al. 2006). In this line, Abdullah and Abdel Hafes ( 2004) reported comparable crude protein and energy contents of PJP to barley grain. Further, these pods contain good amount of protein and energy with 20% soluble sugars and hence are sweet in taste and increase palatability of feed (Talpada et al. 2003). Talpada et al. ( 2002) also reported successful inclusion of PJP at 30% level in compounded cattle feed without any adverse effect on feed intake, digestibility, rumen fermentation. However, the information on feeding of PJP in place of concentrate mixture is scanty in sheep feeding practice. The present study was, therefore, undertaken to evaluate the effect of feeding PJP in replacement of concentrate mixture along with Cenchrus grass (CG) on nutrient utilization and rumen fermentation characteristics in sheep.

## Materials and methods

### Animals and housing

The experimental animals were housed in a well-ventilated enclosure with facility for individual feeding and watering. Animal care, handling and sampling procedures were approved by Institute’s Animal Ethics Committee.

### Feeds and feeding

Twenty four adult Malpura rams (3–5 years, 45.4 ± 4.26 kg) were divided randomly into 3 equal groups (n = 8) of diets: 0 (G1), 30 (G2) and 40% (G3) *Prosopis juliflora* pods (PJP) in replacement of the concentrate mixture. The composition of the concentrate mixture was 67, 30, 2.0, 0.95 and 0.05% of barley, groundnut cake (solvent extracted), mineral mixture, common salt and vitamin supplements, respectively. The PJP was collected, sun dried, ground and then included in the concentrate. The concentrate was fed at 1% live weight while the CG was offered *ad libitum* at 09:00 h. Ad libitum clean drinking water was made available to all the animals. The feeds offered and the residues left were recorded daily.

### Metabolism trial

A metabolism trial was conducted after 30 d of experimental feeding and the study lasted for 10 d (i.e. 4 d adaptation in metabolism cages (5.5’ × 2.0’) followed by 6 d of sample collection). Daily feed intake and output of faeces and urine was recorded and representative samples were collected for further processing. Faeces and urine were collected using a total collection method in which urine was collected into containers with toluene as preservative. Fractional aliquot of feed, faeces and urine samples were brought to laboratory for further processing. The dry matter (DM) of feed, faeces and residue was determined by drying to a constant weight in a forced air oven (NSW-143, Narang Scientific Works Ltd., New Delhi) at 70°C. The dried samples of each day collections were pooled for 6 d, ground to pass a 1 mm screen and preserved for chemical analysis. For N estimation, aliquot of faeces (0.1%) and urine (1%) from the individual animals were collected every morning and pooled in a 500 ml Kjeldahl flask containing 25 ml of concentrated sulphuric acid.

### Nutrient and nutritive value assay

Samples of feeds, residue and faeces were analysed for ash and crude protein (CP) according to procedure of AOAC ( 1995). Neutral detergent fibre (NDF), acid detergent fibre (ADF), acid detergent lignin (ADL) analysed as per Van Soest et al. ( 1991). Organic matter (OM), cellulose and hemicelluloses contents were calculated by difference (i.e., by subtracting ash from DM, ADL from ADF and ADF from NDF, respectively).

The nutritive value of feed was assessed from metabolizable energy (ME) and relative feed value (RFV) of feed in different dietary groups by applying the following equations:

(ARC 1990).

(Jeranyama and Garcia 2004).

### Ruminal attributes

Rumen liquor (50 ml) was collected from each intact rams at 0, 4 and 8 h of post feeding using a stomach tube consecutively for two days. Each sample was placed in a 100 ml glass jar and recorded pH using a portable calibrated digital pH meter (Model PH5652A, Electronic Corporation of India Ltd) within 4–5 min of sampling. Rumen fluid was strained through a four layers of muslin cloth, pooled over two days and stored at -20°C for further analysis, i.e., total N (AOAC 1995) and total volatile fatty acids (VFA) (Barnett and Reid 1957).

### Statistical analysis

The statistical software SPSS version 16.0 (SPSS Inc. Chicago, IL 60606–6307, USA) was used for the analysis of data. Two-way analysis of variance was employed for analyzing the data on rumen parameters to see the effect of treatments, periods and their interactions. Polynomial contrast was applied to assess the effect of levels of supplementation and the significance amongst the means was tested by applying Duncan’s Multiple Range Test and significance was declared at P < 0.05.

## Results

### Nutrient composition, intake and digestibility

The concentrate mixture had CP 225 g/kg DM, while that of PJP was 183 g/kg DM, which also had low ash and lignin content. Unlike, the CG had low CP and high NDF and ADF contents (Table 1). No animals had residual concentrates that was given at 1% of their body weight. Above all, there was no differences (P ≥ 0.59) in roughage, total feed and nutrient intake between the diets (Table 2). Similarly, the digestibility of nutrients did not differ (P ≥0.30) between the diets.

**Table 1:** **Chemical composition (g/kg DM) of feedstuffs fed to rams**

Attributes	Concentrate mixture*	***Prosopis juliflora*** pods	Cenchrus grass
Ash	117	78.0	110
Organic matter	883	922	890
Crude protein	225	183	85.3
Neutral detergent fiber	291	391	717
Acid detergent fiber	133	276	442
Acid detergent lignin	35.8	61.6	66.6
Hemicellulose	158	115	276
Cellulose	97.0	215	375

*Concentrate mixture (% composition): barley 67, groundnut cake (solvent extracted) 30, mineral mixture (calcium 320, phosphorus 62, Manganese 2.7, zinc 2.6, iron 1.0, fluorine 0.9, iodine 0.1, copper 0.1 g/kg) 2, common salt 0.95 and vitamin supplements (Vitamin A 100000 I.U, Vitamin D_3_ 20000 I.U per 100 g) 0.05.

**Table 2:** **Nutrient intake, digestibility, nitrogen balance and ruminal attributes of sheep on different diets**

Parameters	G1	G2	G3	SEM	'P’ value	Significance	
						Linear	Quadratic
***Feed and nutrient intake (g/d)***							
Roughage DM (g/d)	1026	1026	1061	34.3	0.721	0.486	0.699
Concentrate DM (g/d)	408	419	434	14.2	0.456	0.222	0.898
Total DM (g/d)	1434	1445	1495	43.6	0.591	0.343	0.729
Roughage % in diet	71.5	71.0	70.9	0.66	0.822	0.561	0.843
Organic matter	1286	1297	1342	39.0	0.575	0.328	0.735
Crude protein	179	173	175	51.6	0.658	0.551	0.497
Neutral detergent fibre	854	875	912	27.3	0.349	0.160	0.818
Acid detergent fibre	507	524	564	16.4	0.079	0.031	0.563
Hemicellulose	347	352	348	10.9	0.952	0.952	0.763
Cellulose	424	440	460	13.8	0.223	0.090	0.913
Metabolizable energy (MJ)	11.24	11.56	12.01	0.450	0.493	0.246	0.908
***Digestibility (g/kg)***							
Dry matter	561	570	572	16.7	0.896	0.664	0.883
Organic matter	562	571	575	16.4	0.832	0.563	0.889
Crude protein	804	813	804	12.2	0.845	0.980	0.570
Neutral detergent fibre	461	482	495	21.7	0.556	0.294	0.867
Acid detergent fibre	452	475	499	20.6	0.304	0.131	0.997
Hemicellulose	561	560	590	19.4	0.486	0.313	0.527
Cellulose	506	534	525	19.2	0.696	0.578	0.529
***Nitrogen (N) balance***							
N intake (g/d)	28.72	27.65	28.00	0.825	0.658	0.551	0.497
Faecal N (g/d)	5.64	5.18	5.49	0.389	0.707	0.790	0.439
Urinary N (g/d)	8.27	8.26	9.86	0.543	0.097	0.062	0.249
N retained (g/d)	14.81	14.21	12.66	0.645	0.090	0.036	0.556
N retained % of intake	51.4^b^	51.5^b^	45.2^a^	1.70	0.033	0.023	0.148
N retained % of absorbed	64.0^b^	63.4^b^	56.2^a^	2.00	0.031	0.017	0.207
***Ruminal attributes***							
pH	6.34	6.31	6.23	0.061	0.085	0.190	0.767
Total volatile fatty acids (mM/L)	95.0	100.8	99.2	6.05	0.487	0.621	0.615
Total N (g/L)	2.68	2.90	2.96	0.019	0.202	0.149	0.635

Means bearing different superscripts in a row differ significantly (P < 0.05).

G1 (Control), Concentrate + Cenchrus; G2, Concentrate with 30% *P. juliflora* pods + Cenchrus; G3, Concentrate with 40% *P. juliflora* pods + Cenchrus.

### Nitrogen balance

Similar to CP intake, N intake did not differ between the groups (Table 2). A linear decrease (P < 0.05) in N retention was detected with the increase of PJP in the diet. Similar trend was also observed for N retention as per cent of intake or absorbed. However, no difference (P > 0.05) was noticed in N balance between G1 and G2 diets.

### Ruminal attributes

**Figure 1:**
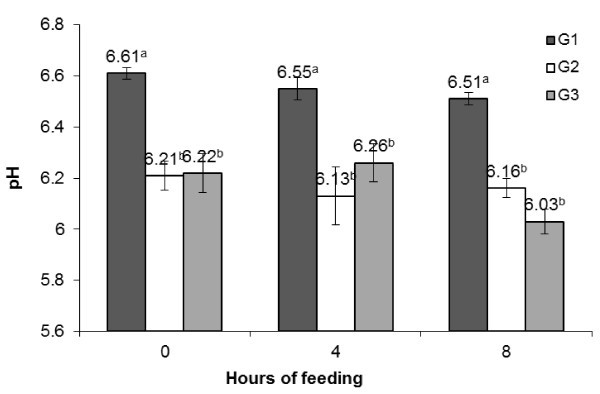
**Diurnal changes in rumen pH during post-feeding hours in different groups.**

**Figure 2:**
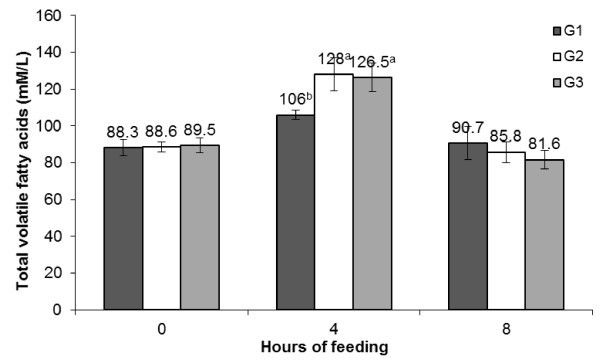
**Diurnal changes in rumen volatile fatty acids (mM/L) during post-feeding hours in different groups.**

**Figure 3:**
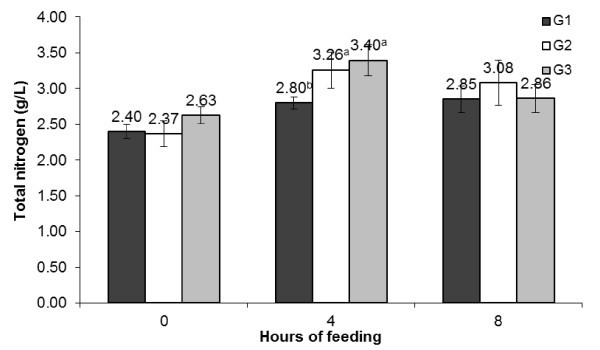
**Diurnal changes in rumen total N during post-feeding hours in different groups.**

There was a decline in pH in G2 and G3 compared to G1 during both pre- and post-feeding periods (Figure 1). The post-prandial alterations in rumen pH were also significant within the groups. Total VFA concentration showed periodic fluctuation with higher values in all the groups during 4 h post-feeding that dropped during 8 h to comparable values as it was during pre-feeding (Figure 2). No difference was observed in total VFA concentration between the groups, but the values at 4 h post-feeding was significantly higher in PJP fed groups. The total N concentration showed a similar trend (Figure 3) as it was for total VFA in different dietary groups as well as during post-feeding hours.

### Nutritive value of diets and nutritional profile of sheep

There was comparable digestible OM and ME content of total diets that were being fed to animals under different groups except that the CP and DCP content declined linearly in G2 and G3 compared to G1 (Table 3). However, the DM, OM and ME intake per unit LW did not differ between the groups. Inclusion of PJP in the diet did not have any significant (P > 0.05) effect on alteration in LW during this study.

**Table 3:** **Nutritive value of diets and nutritional profile of sheep in different groups**

Parameters	G1	G2	G3	SEM	'P’ value	Significance	
						Linear	Quadratic
***Nutritive value***							
Digestible organic matter (g/kg)	504	513	517	14.7	0.209	0.395	0.023
Crude protein (g/kg)	125.2^b^	119.5^ab^	117.1^a^	0.79	<0.001	<0.001	0.116
Digestible crude protein (g/kg)	100.6^b^	97.2^ab^	94.2^a^	1.50	0.033	0.010	0.894
Metabolizable energy (MJ/kg)	7.85	7.99	8.05	0.229	0.814	0.542	0.882
Relative feed value*	149	150	147	5.1	0.904	0.752	0.758
***Nutrient intake (g/kg LW)***							
Dry matter	34.2	34.0	33.2	0.70	0.561	0.311	0.757
Digestible organic matter	17.3	17.4	17.1	0.58	0.932	0.860	0.748
Crude protein	4.28^b^	3.95^a^	3.88^a^	0.059	0.002	<0.001	0.740
Digestible crude protein	3.45^b^	3.20^ab^	3.12^a^	0.072	0.026	0.008	0.873
Metabolizable energy (J)	269	271	267	9.1	0.932	0.860	0.748
***Nutrient intake (g/kgW*** ^***0.75***^ ***)***							
Dry matter	87.0	86.7	86.0	1.62	0.890	0.643	0.920
Digestible organic matter	43.9	44.5	44.3	1.43	0.853	0.825	0.834
Crude protein	10.90^b^	10.36^a^	10.06^a^	0.139	0.004	0.001	0.508
Digestible crude protein	8.76	8.42	8.09	0.174	0.055	0.018	0.980
Metabolizable energy (J)	684	693	691	22.9	0.953	0.825	0.834
***Live weight change***							
Initial live weight (kg)	45.3	45.2	45.8	1.57	0.963	0.842	0.853
Final live weight (kg)	45.5	46.2	46.5	1.59	0.895	0.646	0.939
Gain (kg)	0.18	0.99	0.78	0.430	0.399	0.335	0.342

ME intake (MJ/kg DM) = [(digestible OM, g/kg DM)/1000] × 18.5 × 0.81; {ARC, 1990}.

* Relative feed value (RFV) = {Digestible dry matter (%) × Dry matter intake (% of live weight)}/1.29; {Jeranyama and Garcia, 2004}.

Means bearing different superscripts in a row differ significantly (P < 0.05).

G1 (Control), Concentrate + Cenchrus; G2, Concentrate with 30% *P. juliflora* pods + Cenchrus; G3, Concentrate with 40% *P. juliflora* pods + Cenchrus.

## Discussion

The nutrient composition of CG was in close agreement to earlier reports (Mishra et al. 1997; Shinde et al. 1997). With 18.34% CP, PJP can be grouped in protein-rich concentrates and the findings corroborated with earlier reports (Sawal et al. 2004; Mahgoub et al. 2005). Further, concentrates replaced by PJP at 30 and 40% showed comparable acceptability as evidenced from feed intake in different groups. A non-significant difference in digestibility of nutrients between the groups corroborate to this level of PJP inclusion as an alternate feed resource. Rao and Reddy ( 1983) found similar effect on feed intake in cattle fed concentrate mixtures containing 40% PJP and so was the observation up to 20% of the diet by Talpada et al. ( 2002). On the basis of total feed intake, the proportion of PJP in the total diets was only 9 and 12%, which was considerably less than these reported inclusion levels and thus the diets were readily accepted by sheep and there was comparable DM intake in the three dietary groups.

A similar intake and digestibility of nutrients between the groups contributed to comparable digestible OM and ME contents in the three diets. But, a reduced level of CP and DCP content of diet in G2 and G3 was attributable to relatively lower CP in PJP compared to concentrate mixture (Table 1) when replaced at 30 and 40% level. Contrast to the findings in the present experiment, a great degree of variability was noted in digestibility of PJP (Sawal et al., 2004) and it may be attributed to variability in topography and season of collection (Chopra and Hooda 2001). Reports on the composition and nutritive value of PJP showed that they are a potential source of protein and energy, although pod composition varies with location. The RFV of feeds calculated from actual values (in place of predicted values) could be an indicator to compare the three diets, and a value of 150 may thus be considered to support animals above its maintenance requirement if intake is not limited for production output. This can be further substantiated by assessing the intake of CP, DCP and ME per kg W^0.75^ in different groups, which showed that all the three diets could very well support production due to additional 30% ME available from the respective diets over and above the maintenance need (NRC 2007). The level of CP/DCP in the three diets was 50% higher and could thus very well support requirement of growing animals or the extra amount can be excluded to reduce the feed cost of diet in the feeding of sheep. The N balance data that showed a decline in N utilization (N retained, N retained as percent of intake and absorbed) at 40% level of PJP inclusion could be attributed to relatively higher (P = 0.097) urinary excretion of N. Moreover, a significant amount of N retention that was not reflected with additional LW gain in mature Malpura sheep can be harnessed for growth and production in desired animals.

The effect replacing concentrate mixture with PJP on ruminal pH, total VFA (mM/L) and total N (g/L) was assessed to take note of change in ruminal environment. A reduction in pH and increase in total VFA at 4 h post-feeding in G2 and G3 compared to G1 may be attributed to presence of fermentable carbohydrates in PJP. It has been reported that PJP is rich in saccharose (20-25% of DM) and reduced sugar (10-20% of DM) (Silva 1986). Further, a corresponding increase in total N could be correlated to higher concentration of soluble N in PJP and this might have contributed to higher urinary output. Moreover, PJP with ready supply of fermentable carbohydrate in the rumen might have improved utilization of ammonia by microbes and ensured better supply of protein and energy for production. The present findings on rumen fermentation are in close agreement to those reported earlier (Ravikala et al. 1993; Talpada et al. 2002; Sharma et al. 2007).

On the basis of this study on intake and digestibility of nutrients, rumen fermentation attributes and nutritional profile of Malpura sheep, it may be suggested that complete rations using non conventional feed ingredients could be successfully made and tried in the diet of sheep to bridge the gap between demand and supply and to get maximum economic production. Feed costs were reduced by 26% when PJP replaced up to 50% of the concentrate diet of sheep, without affecting their growth (Sawal et al. 2004). The local price of PJP is observed to be 25% of the price of concentrate mixture and thus at 40% level of replacement the price would be reduced by 30%. In a series of experiments, Talpada et al. ( 2002) also found suitability of incorporating PJP in the complete diet of crossbred calves. Thus, PJP that is available at ease with the farmers of semi-arid/arid tropics could very well substitute cereal grains and oil cakes in the concentrate mixture. This would encourage the incorporation of unutilized or underutilized feeds and thereby harnessing and optimizing maximum benefit from the available natural resources for sustainability.

## Conclusion

It is concluded that *Prosopis juliflora* pods can replace concentrate mixture up to 40% in sheep feeding without having any adverse effect on nutrient intake and utilization as well as on ruminal attributes. Thus, PJP that is available at ease with the farmers of semi-arid/arid tropics could very well substitute cereal grains and oil cakes in the concentrate mixture and may thus provide opportunity to economize the ration for sheep while sparing these ingredients for poultry and other livestock or food for human consumption.
